# Supplementary material for: Multiscale model of defective interfering particle replication for influenza A virus infection in animal cell culture
Source: PLoS Comput Biol. 2021 Sep 7;17(9):e1009357. doi: 10.1371/journal.pcbi.1009357 (PMC8448327; doi:10.1371/journal.pcbi.1009357)
Supplement: S1 Appendix — (DOCX) [file pcbi.1009357.s001.docx]

**Intracellular model**

The intracellular model is based on a model developed in [1]. Eqs (S18)-(S19), (S27)-(S28) and (S41)-(S46) were introduced or modified for this publication.

**Virus entry**

with $B_{\text{n}}=B_{\text{n}}^{\text{Tot}}-V_{\text{n}}^{\text{Att}}-D_{\text{n}}^{\text{Att}},\text{ n}\in\left\{\text{Hi},\text{Lo}\right\}$

and $k_{\text{n}}^{\text{Dis}}=\frac{k_{\text{n}}^{\text{Att}}}{k_{\text{n}}^{\text{Eq}}}$

with

$$k_{\text{En}}^{\text{Deg}}=\frac{1-F_{\text{Fus}}}{F_{\text{Fus}}}k^{\text{Fus}},\text{ }0<F_{\text{Fus}}\leq1$$

$V^{\text{Ex}}$ and $D^{\text{Ex}}$ denote extracellular standard viruses (STVs) and defective interfering particles (DIPs), respectively, that bind to free binding sites $B_{\text{n}}$ (sialic acid residues) on the cell membrane. According to [2], we distinguish between high-affinity (n = hi) and low-affinity (n = lo) binding sites. Virus particles attached to binding sites ($V_{\text{n}}^{\text{Att}}$,$D_{\text{n}}^{\text{Att}}$) can either dissociate from the cell surface with rate $k_{\text{n}}^{\text{Dis}}$ or undergo receptor-mediated endocytosis. Subsequently, enveloped virus particles ($V^{\text{En}}$,$D^{\text{En}}$) either fuse with the endosomal membrane transferring their viral genome into the cytoplasm or are degraded in lysosomes.

**Virus replication**

with

$$k_{\text{V}}^{\text{Syn}}=\frac{K_{\text{V}}}{f_{\text{D,V}}(t_{\text{I}})}\text{ }$$

and

with

i = 1,…,9 ; j = 1,…,8; and k = 2,…,8

After the viral ribonucleoproteins (vRNP [$V^{\text{Cyt}}$,$D^{\text{Cyt}}$]) enter the cytoplasm they can get imported into the nucleus. There, the vRNPs for different segments of the viral genome ($Vp_{\text{i}}^{\text{Nuc}}$) are used as templates for viral replication, with i = 1,…,8 denoting the eight regular full-length (FL) segments and i = 9 representing the defective interfering (DI) segment. In the nucleus, complementary RNA (cRNA [$R_{\text{i}}^{\text{C}}$]) is transcribed from the templates and subsequently stabilized by binding viral RNA-dependent-RNA-polymerase (RdRp [$P_{\text{RdRp}}$]) to form $R_{\text{RdRp,i}}^{\text{C}}$ and binding nucleoproteins (NP [$P_{\text{NP}}$]) to form complementary ribonucleoproteins (cRNP [$Cp_{\text{i}}$]). Non-stabilized cRNA is highly susceptible to degradation compared to cRNP ($k_{\text{R}}^{\text{Deg}}$>$k_{\text{RRdRp}}^{\text{Deg}}$>$k_{\text{Rnp}}^{\text{Deg}}$). We assume that DI cRNA shows an advantage in replication compared to FL cRNA, which is implemented in Eq (S16) via the advantage factor $F_{\text{Adv}}$. The next step of replication is the transcription of intracellular viral RNA (vRNA) from the cRNP templates. The newly generated vRNA ($R_{\text{i}}^{\text{V}}$) is also stabilized by binding RdRp and NP forming $R_{\text{RdRp.i}}^{\text{V}}$ and $Vp_{\text{i}}^{\text{Nuc}}$, respectively. Here, we implemented the assumption that the rate of vRNA transcription in Eq (S17) is reduced depending on the ratio of DIP to STV concentration $f_{\text{D,V}}(t_{\text{I}})$ at the time point of cell infection $t_{\text{I}}$. This reduction only occurs when a minimum DIP concentration of $F_{\text{MODIP}}$ is surpassed.

The viral matrix protein 1 (M1) can also bind to vRNPs in the nucleus to form $Vp_{\text{M1,i}}^{\text{Nuc}}$, which is replication incompetent. In this state, the nuclear export protein (NEP [$P_{\text{NEP}}$]) can bind to vRNP, which enables its export into the cytoplasm. After leaving the nucleus the vRNP-M1-NEP complex is referred to as $Vp_{\text{M1,i}}^{\text{Cyt}}$.

**Viral transcription and protein synthesis**

$$\frac{dR_{\text{i}}^{\text{M}}}{dt}=f_{\text{M}}\frac{k_{\text{M}}^{\text{Syn}}Vp_{\text{i}}^{\text{Nuc}}}{L_{\text{i}}\left(1+\frac{P_{\text{Rdrp}}}{K_{\text{R}}}\right)}-k_{\text{M}}^{\text{Deg}}R_{\text{i}}^{\text{M}}$$

with

While in the nucleus, vRNPs are also templates for the transcription of viral mRNA ($R_{\text{i}}^{\text{M}}$) required for viral protein synthesis. Each of the eight FL genome segments i encodes for different viral mRNAs. The transcription of viral mRNAs is assumed to be dependent on their respective length ($L_{\text{i}}$) and is reduced by increasing amounts of free RdRp [3, 4]. Additionally, we incorporated the assumption that RdRp-related segments, i.e., FL segment 1 to 3 as well as the DI segment, are transcribed at a lower rate based on the factor $F_{\text{M}}$. Degradation of viral mRNAs occurs with the rate $k_{\text{M}}^{\text{Deg}}$ and they are translated into viral proteins $P_{\text{i}}$ in the cytoplasm. The three polymerase sub-unit proteins ($P_{\text{PB1}}$, $P_{\text{PB2}}$ and $P_{\text{PA}}$) unite to form the RdRp, which is required at various steps of virus replication. The viral mRNA of segment 7 can be translated into two different proteins, i.e., M1 and M2, and the ratio of their production is defined by the parameter $F_{\text{Spl7}}$. Besides their role in viral replication, the proteins M1, M2, hemagglutinin (HA) and neuraminidase (NA) are also necessary for virion release, because they perform structural functions in the progeny virus particle.

**Complex formation and virus particle release**

with

$$\text{j}\in\left\{\text{HA,NA,M1,M2}\right\}$$

To describe the packaging of vRNPs in the cytoplasm, a segment-specific mechanism is used. Therefore, the progeny genomes form complexes including a single copy of each segment. Both STV and DIP complexes ($V_{\text{Cplx}}^{\text{Cyt}}$, $D_{\text{Cplx}}^{\text{Cyt}}$) contain FL segments 2 to 8 and additionally either FL or DI segment 1. Complexes are formed with the rate $k^{\text{Cplx}}$ and can get degraded. The release of packed STV and DIP complexes is described via the rates $r_{\text{STV,Tot}}^{\text{Rel}}$ and $r_{\text{DIP,Tot}}^{\text{Rel}}$, respectively. We differentiate between the total release of virus particles indicated by these rates and that of fully functional STVs and DIPs ($r_{\text{STV}}^{\text{Rel}}$, $r_{\text{DIP}}^{\text{Rel}}$). All release rates are calculated by considering the maximum release rate $k^{\text{Rel}}$, the abundance of required viral proteins and the availability of virus complexes. The percentage of fully functional STVs and DIPs that are produced is determined by the variable $F_{\text{Par}}$, which decreases over time of infection.

**Primary mRNA in DIP-only infected cells**

$$\frac{dR_{\text{i,}I_{\text{DIP}}}^{\text{M}}}{dt}=f_{\text{M}}\frac{k_{\text{M}}^{\text{Syn}}}{L_{\text{i}}}\frac{D(t_{\text{I}})}{C_{\text{Tot}}(t_{\text{I}})}-k_{\text{M}}^{\text{Deg}}R_{\text{i,}I_{\text{DIP}}}^{\text{M}}\text{ , i}=2,...,9$$

Virus replication in cells only infected by DIPs is disregarded, except for the transcription of viral mRNA from vRNP templates. Here, the MODIP at the time of infection $\frac{D(t_{\text{I}})}{C_{\text{Tot}}(t_{\text{I}})}$ determines the amount of vRNP templates provided initially. As no progeny RdRp can be produced due to the missing FL segment 1, viral mRNA transcription is not affected by the effects described in [3, 4]. Nevertheless, the generation of RdRp-related mRNAs is still reduced and viral mRNA degradation occurs.

**Cell population model**

The cell population model is based on a multiscale model developed in our group [5] using the standard cell population balance and an expansion introduced in [6] to adjust it for IAV infection of cell cultures. We implemented various DIP-related populations to account for their propagation and effect on STV production.

**Cell populations**

with

$$\mu(t)=\left(f_{\mu}\frac{\mu_{\text{Max}}}{T_{\text{Max}}}\left[T_{\text{Max}}-C_{\text{Tot}}(t)\right]\right)_{+}$$

and

with

The cell population model describes populations of uninfected cells (*T*), apoptotic uninfected cells (*T*_A_), STV-only infected cells (*I*_STV_), DIP-only infected cells (*I*_DIP_), co-infected cells (*I*_CO_), and apoptotic infected cells (*I*_A_). Uninfected cells and DIP-only infected cells can grow with rate *μ*, which is reduced by the factor $F_{\mu}$ if the combined MOI and MODIP exceed a value of 6. Additionally, they can get infected by STVs with rate $r_{\text{STV}}^{\text{Inf}}$ and undergo apoptosis with rate $k_{\text{T}}^{\text{Apo}}$. DIPs can infect uninfected cells and STV-only infected cells with rate $r_{\text{DIP}}^{\text{Inf}}$. STV-only infected cells and co-infected cells get apoptotic with an increased rate of virus-induced apoptosis $k_{\text{I}}^{\text{Apo}}(\tau)$. This rate depends on the infection age *τ* and utilizes a logistic function, which can approximate the cumulative density function of the normal distribution [7]. To this end, the parameter $v_{\text{Apo}}$, which affects the steepness of the increase of the apoptosis rate over time, was fixed to a value of 1.7 as this represents a normal distribution most closely. All apoptotic cells are lysed with rate $k_{\text{Lys}}$.

**Age-segregated infected cell populations**

The populations for STV-only infected cells and co-infected cells are segregated by their infection age *τ* according to [6]. Upon STV infection of uninfected cells at time $t-\tau$, STV-only infected cells with age zero $I_{\text{STV}}(t,0)$ emerge and are subject to further infection by DIPs or virus induced apoptosis. Co-infected cells $I_{\text{CO}}(t,\tau)$ are created when an STV-only infected cell is infected by a DIP or a DIP-only infected cell is infected by a STV and can get apoptotic with rate $k_{\text{I}}^{\text{Apo}}(\tau)$.

**Virus particle release**

with

$B_{\text{n}}^{\text{V}}=B_{\text{n}}^{\text{Tot}}\left(T+T_{\text{A}}+I_{\text{DIP}}\right)-V_{\text{n}}^{\text{Att}}$ ,

,

and

Infectious STVs (*V*) and DIPs (*D*) in the extracellular space attach to high- and low affinity receptors. For the calculation of virus binding, we differentiate between receptors that STVs can attach to ($B_{\text{n}}^{\text{V}}$), i.e., receptors on uninfected and DIP-only infected cells, and their counterpart for DIPs ($B_{\text{n}}^{\text{D}}$), which reside on uninfected and STV-only infected cells. Infectious STVs and DIPs can get degraded over time with the rates $k_{\text{V}}^{\text{Deg}}$ and $k_{\text{D}}^{\text{Deg}}$, respectively, rendering them incapable to infect cells. For simulation $k_{\text{V}}^{\text{Deg}}=k_{\text{D}}^{\text{Deg}}$ was assumed. The rates $r_{\text{i,j}}^{\text{Rel}}(\tau)$ describe virus particle release of $\text{i}\in\text{\{STV, DIP\}}$ from $\text{j}\in\text{\{}I_{\text{STV}}\text{, }I_{\text{CO}}\text{\}}$ cells. Total concentrations of released STVs and DIPs are denoted by $V_{\text{Tot}}$ and $D_{\text{Tot}}$, respectively.

**Virus entry**

with

The entry of virus particles into cells is also described on the population level, because the amount of viruses attached to cells and undergoing endocytosis is used for the simulation of the intracellular model. Free infectious virus particles attach to and dissociate from cells with the rates $k_{\text{c,n}}^{\text{Att}}$ and $k_{\text{n}}^{\text{Dis}}$, respectively. Then, attached virus particles, i.e., $V_{\text{n}}^{\text{Att}}$ and $D_{\text{n}}^{\text{Att}}$, perform receptor-mediated endocytosis with the rate $k^{\text{En}}$. Attached virions are also removed from the population level upon cell infection by either STVs ($r_{\text{STV}}^{\text{Inf}}$) or DIPs ($r_{\text{DIP}}^{\text{Inf}}$) and due to the lysis of cells ($r_{\text{STV}}^{\text{Lys}}$,$r_{\text{DIP}}^{\text{Lys}}$). These first two rates are calculated based on the amount of virions in endosomes ($V^{\text{En}}$,$D^{\text{En}}$) and the latter on the number of apoptotic cells *T*_A_ in relation to the total amount of cells not infected either by DIPs or STVs.

**Supplementary references**

1. Laske T, Heldt FS, Hoffmann H, Frensing T, Reichl U. Modeling the intracellular replication of influenza A virus in the presence of defective interfering RNAs. Virus Research. 2016;213:90-99.
2. Nunes-Correia I, Ramalho-Santos J, Nir S, de Lima MCP. Interactions of influenza virus with cultured cells: Detailed kinetic modeling of binding and endocytosis. Biochemistry. 1999;38(3): 1095-1101.
3. Rodriguez A, Pérez-González A, Nieto A. Influenza virus infection causes specific degradation of the largest subunit of cellular RNA polymerase II. Journal of Virology. 2007;81(10):5315-5324.
4. Martínez-Alonso M, Hengrung N, Fodor E. RNA-free and ribonucleoprotein-associated influenza virus polymerases directly bind the serine-5-phosphorylated carboxyl-terminal domain of host RNA polymerase II. Journal of Virology. 2016;90(13):6014-6021.
5. Rüdiger D, Kupke SY, Laske T, Zmora P, Reichl U. Multiscale modeling of influenza A virus replication in cell cultures predicts infection dynamics for highly different infection conditions. PLOS Comput Biol. 2019;15(2):e1006819.
6. Heldt FS, Frensing T, Pflugmacher A, Gröpler R, Peschel B, Reichl U. Multiscale modeling of influenza A virus infection supports the development of direct-acting antivirals. PLOS Computational Biology. 2013;9(11): e1003372.
7. Bowling SR, Khasawneh MT, Kaewkuekool S, Cho BR. A logistic approximation to the cumulative normal distribution. Journal of Industrial Engineering and Management. 2009;2(1):114-127.
8. Arava Y, Wang YL, Storey JD, Liu CL, Brown PO, Herschlag D. Genome-wide analysis of mRNA translation profiles in Saccharomyces cerevisiae. Proceedings of the National Academy of Sciences of the United States of America. 2003;100: 3889-3894.
9. Heldt FS, Frensing T, Reichl U. Modeling the intracellular dynamics of influenza virus replication to understand the control of viral RNA synthesis. Journal of Virology. 2012;86(15): 7806-7817.
10. Robb NC, Jackson D, Vreede FT, Fodor E. Splicing of influenza A virus NS1 mRNA is independent of the viral NS1 protein. Journal of General Virology. 2010;91: 2331-2340.
11. Babcock HP, Chen C, Zhuang XW. Using single-particle tracking to study nuclear trafficking of viral genes. Biophysical Journal. 2004;87: 2749-2758.
12. Spirin, AS. Ribosome structure and protein biosynthesis. The Benjamin/Cummings Publishing Company. 1986.
13. Lamb RA, Krug RM. Orthomyxoviridae: the viruses and their replication. In: Knipe DM, Howley PM, Griffin EG, editors. Fields virology, 4th edition. Lippincott Williams & Wilkins; 2001. p.1487-153.1
14. Wakefield L, Brownlee GG. Rna-Binding Properties of Influenza-a Virus Matrix Protein M1. Nucleic Acids Research. 1989;17: 8569-8580.
15. Portela A, Digard P. The influenza virus nucleoprotein: a multifunctional RNA-binding protein pivotal to virus replication. Journal of General Virology. 2002;83: 723-734.
16. Schulze-Horsel J, Schulze M, Agalaridis G, Genzel Y, Reichl U. Infection dynamics and virus-induced apoptosis in cell culture-based influenza vaccine production-Flow cytometry and mathematical modeling. Vaccine. 2009;27: 2712-2722.
17. Dimmock NJ, Rainsford EW, Scott PD, Marriott AC. Influenza virus protecting RNA: an effective prophylactic and therapeutic antiviral. Journal of Virology. 2008;82(17):8570-8578.
