# Supplementary figures and images for: Multiscale model of defective interfering particle replication for influenza A virus infection in animal cell culture
Source: PLoS Comput Biol. 2021 Sep 7;17(9):e1009357. doi: 10.1371/journal.pcbi.1009357 (PMC8448327; doi:10.1371/journal.pcbi.1009357)

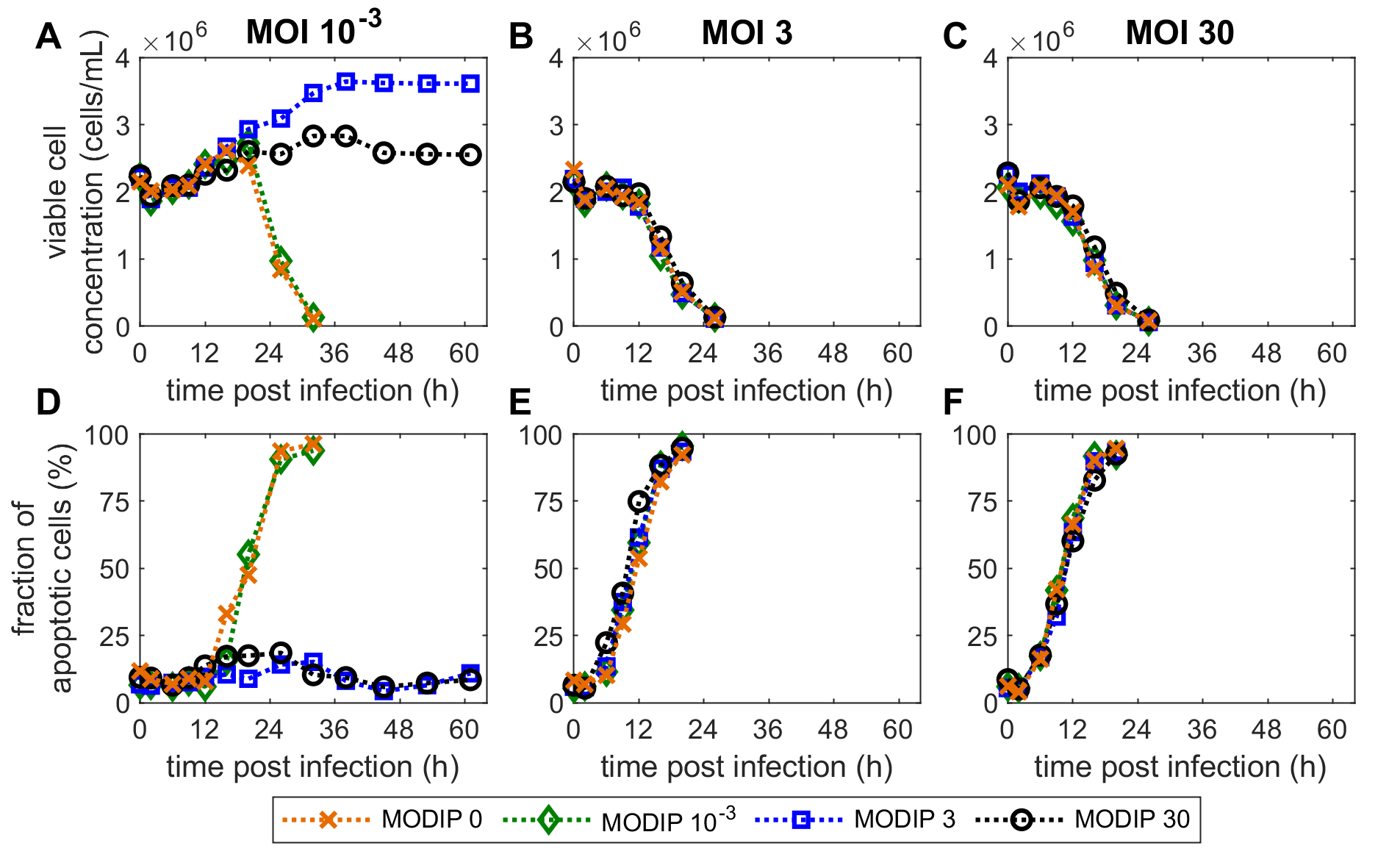

Supplement: S1 Fig — Measurements of (A-C) viable cell concentration and (D-F) the fraction of apoptotic cells for infections with MOI 10−3, 3 and 30 using different MODIPs. (TIF) [file pcbi.1009357.s003.tif]

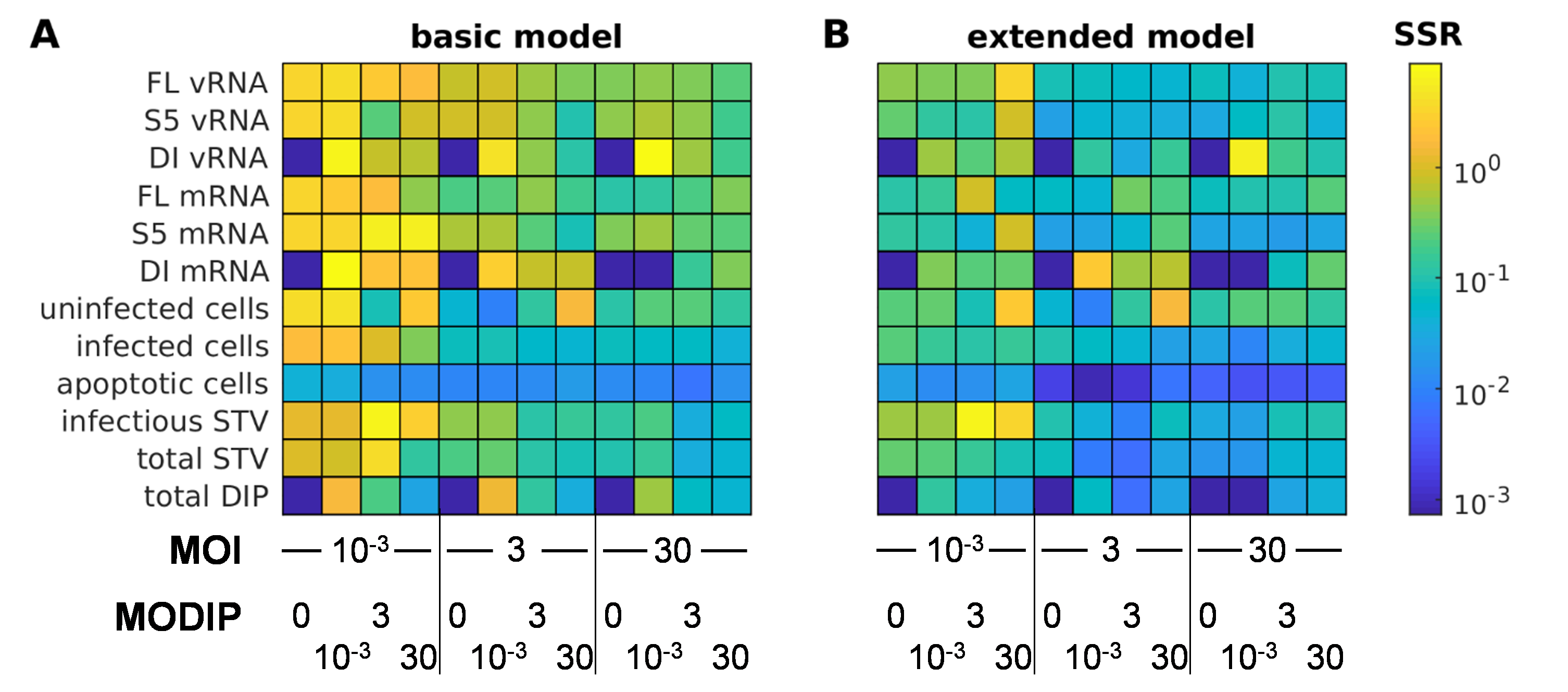

Supplement: S2 Fig — The sum of squared residuals for each individual measured property is depicted. Logarithmic errors of each variable were normalized to the respective maximum measurement value. The (A) basic model and the (B) extended model were calibrated to a wide range of experimental data. Measured properties include vRNA and mRNA of full-length (FL) segment 1, defective-interfering (DI) segment 1 and segment 5 (S5), the concentration of uninfected, infected and apoptotic cells, total and standard virus (STV) titers as well as DIP titers. (TIF) [file pcbi.1009357.s004.tif]

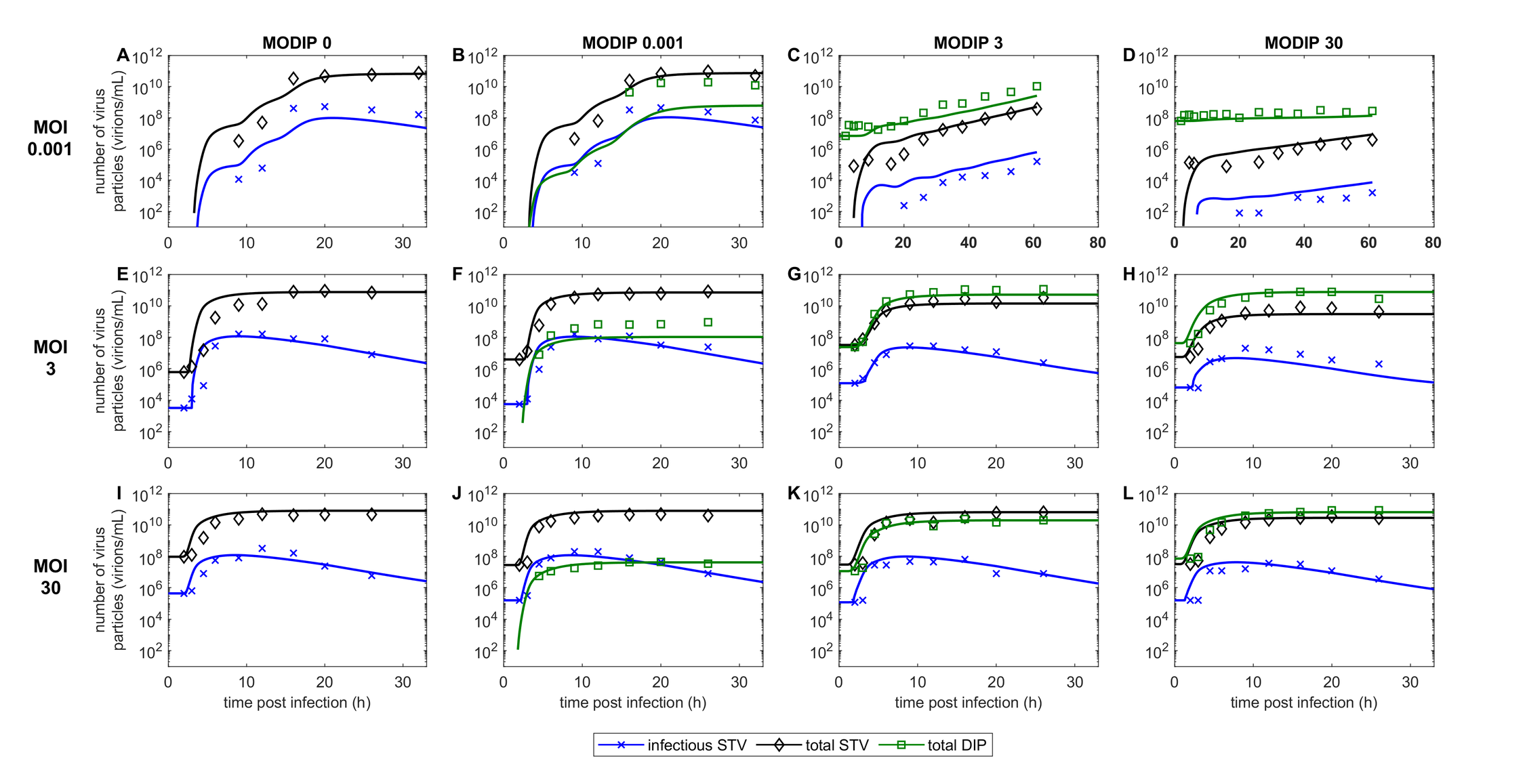

Supplement: S3 Fig — Model fits to measurements of the infectious STV titer, the total amount of STVs and the total amount of DIPs for MDCKsus infections with MOI 10−3, 3 and 30 using different MODIPs. (TIF) [file pcbi.1009357.s005.tif]

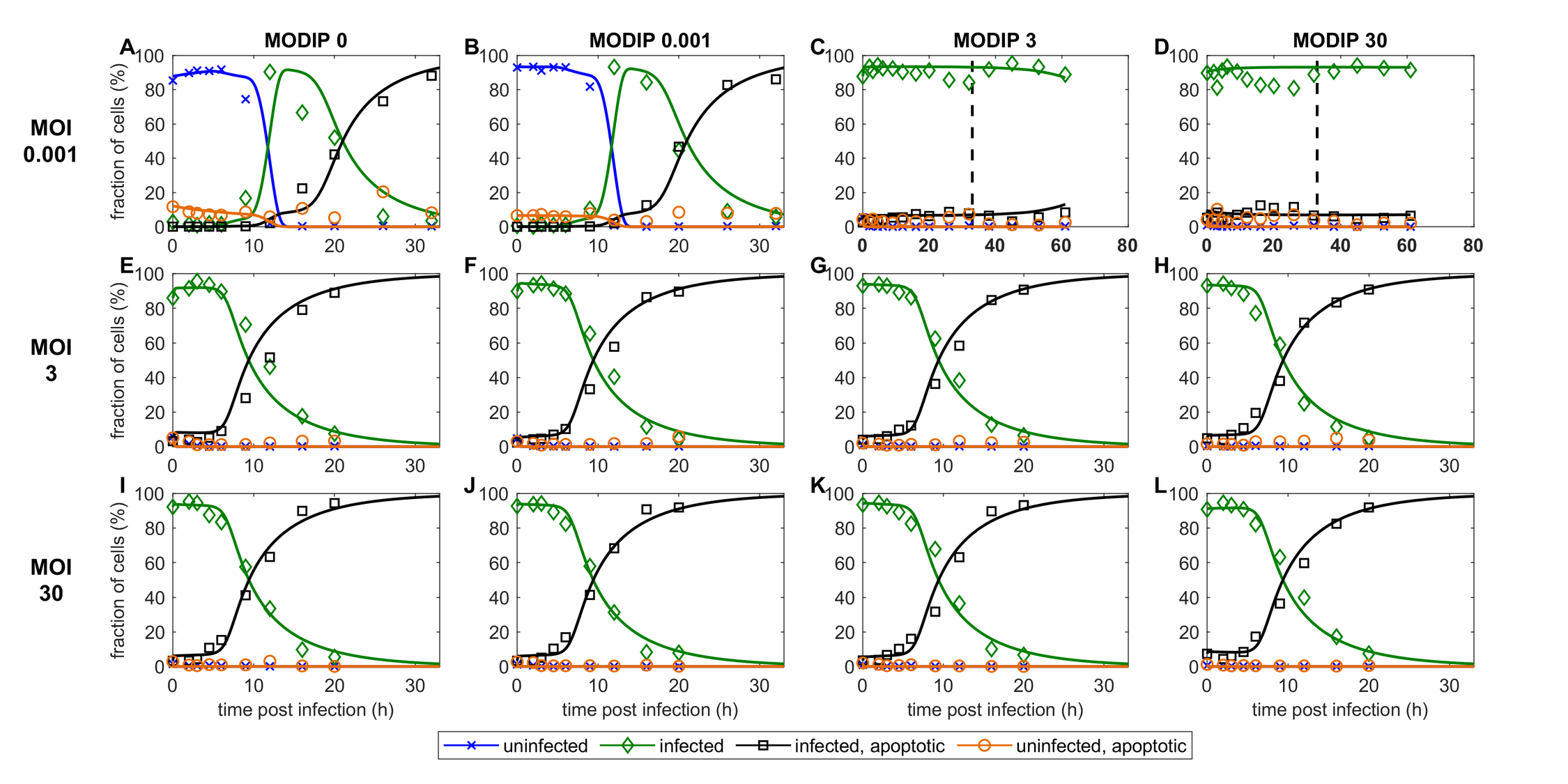

Supplement: S4 Fig — Model fits to measurements of the fraction of uninfected, uninfected and apoptotic, infected, infected and apoptotic cells for MDCKsus infections with MOI 10−3, 3 and 30 using different MODIPs. (TIF) [file pcbi.1009357.s006.tif]

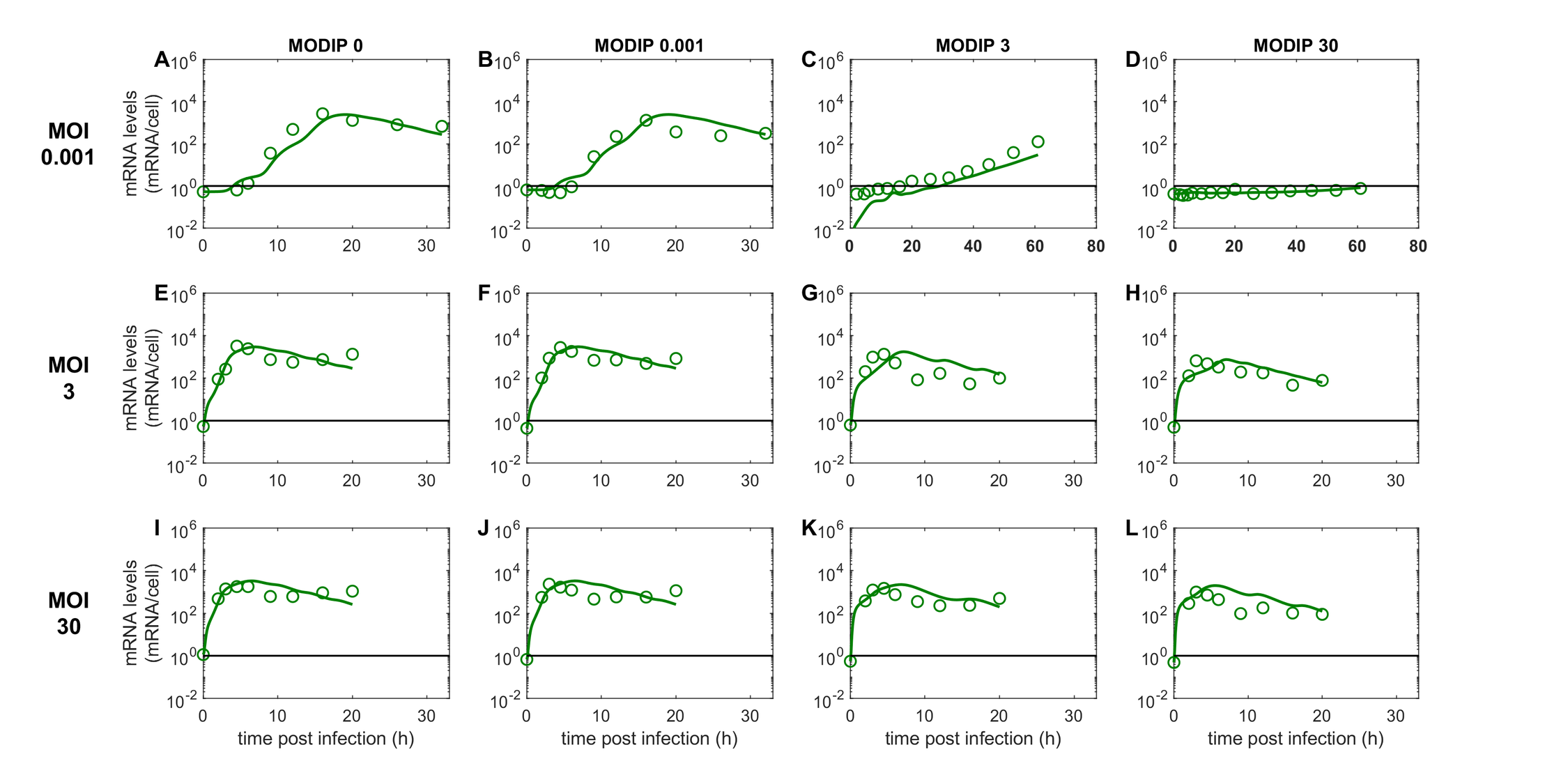

Supplement: S5 Fig — Model fits to measurements of the intracellular levels of FL mRNA for MDCKsus infections with MOI 10−3, 3 and 30 using different MODIPs. (TIF) [file pcbi.1009357.s007.tif]

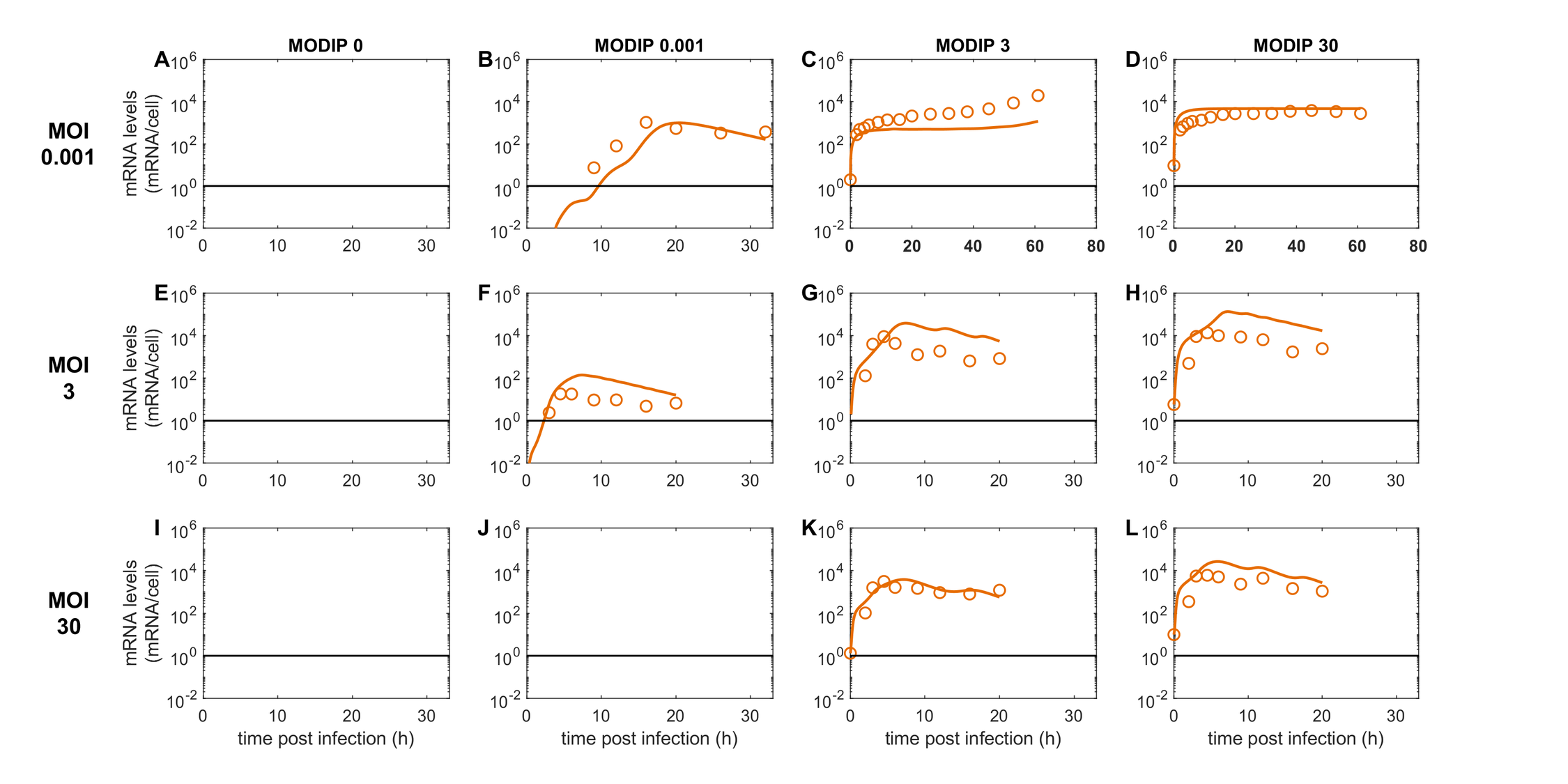

Supplement: S6 Fig — Model fits to measurements of the intracellular levels of DI mRNA for MDCKsus infections with MOI 10−3, 3 and 30 using different MODIPs. (TIF) [file pcbi.1009357.s008.tif]

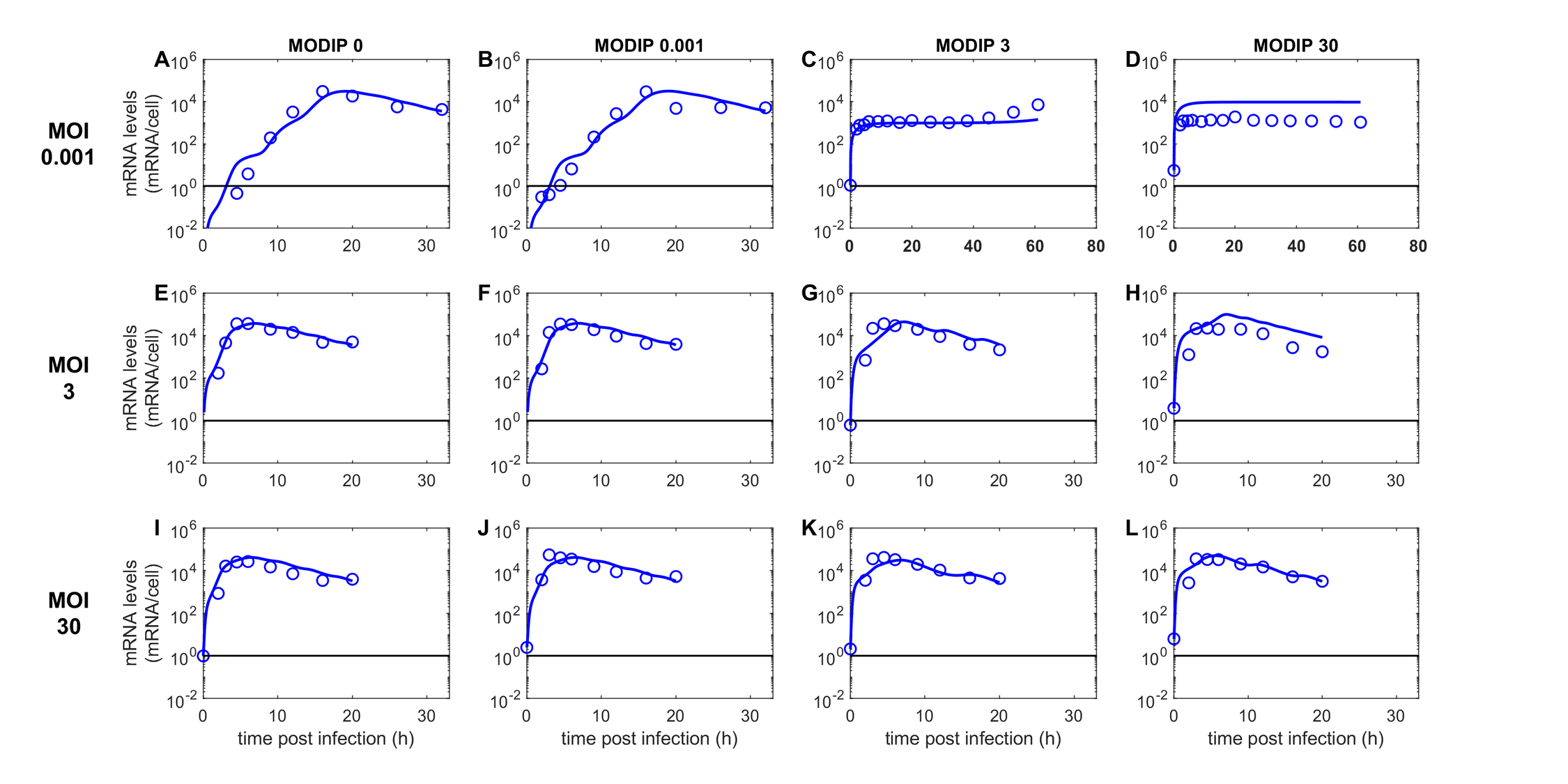

Supplement: S7 Fig — Model fits to measurements of the intracellular levels of segment 5 mRNA for MDCKsus infections with MOI 10−3, 3 and 30 using different MODIPs. (TIF) [file pcbi.1009357.s009.tif]

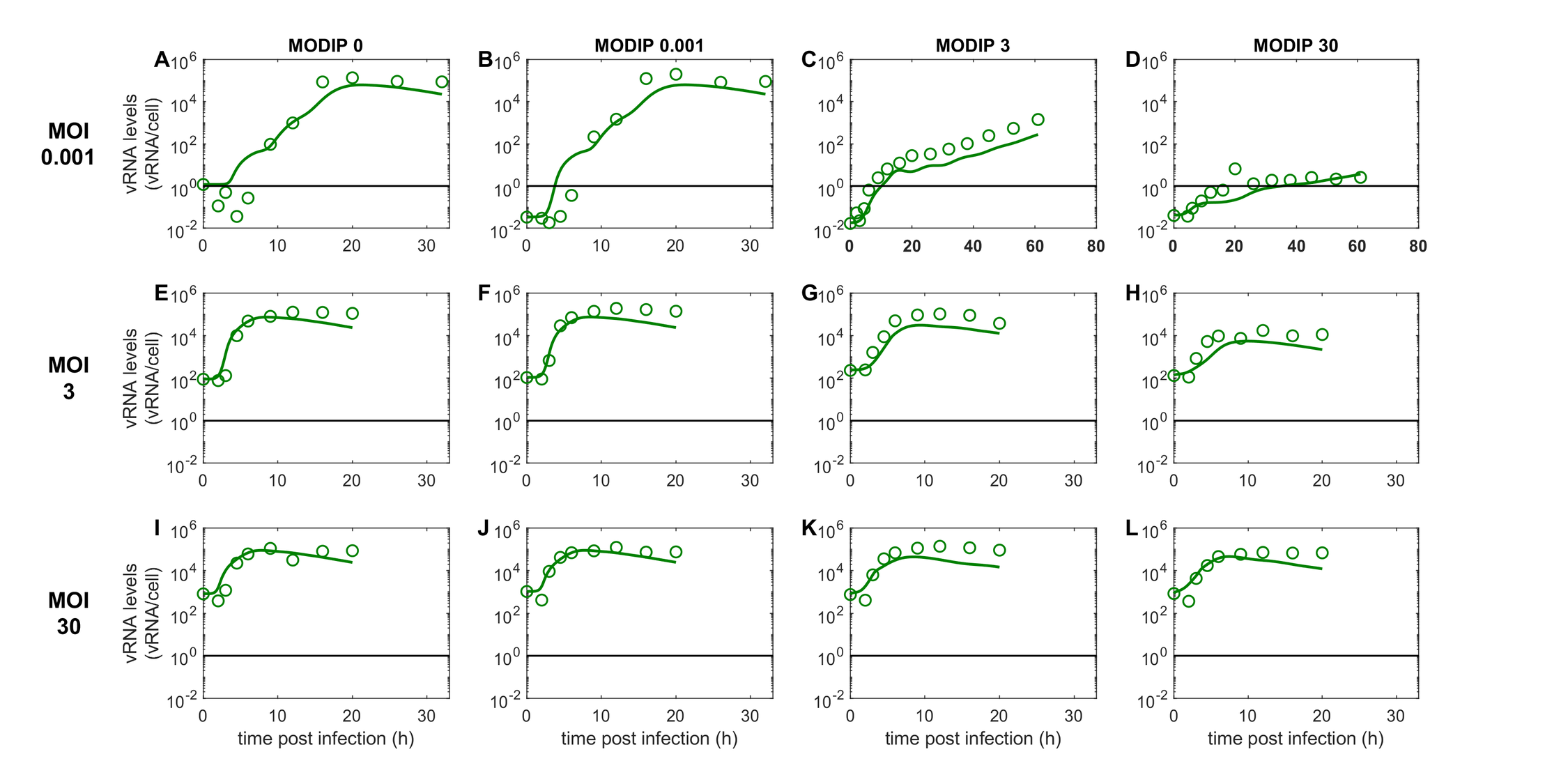

Supplement: S8 Fig — Model fits to measurements of the intracellular levels of FL vRNA for MDCKsus infections with MOI 10−3, 3 and 30 using different MODIPs. (TIF) [file pcbi.1009357.s010.tif]

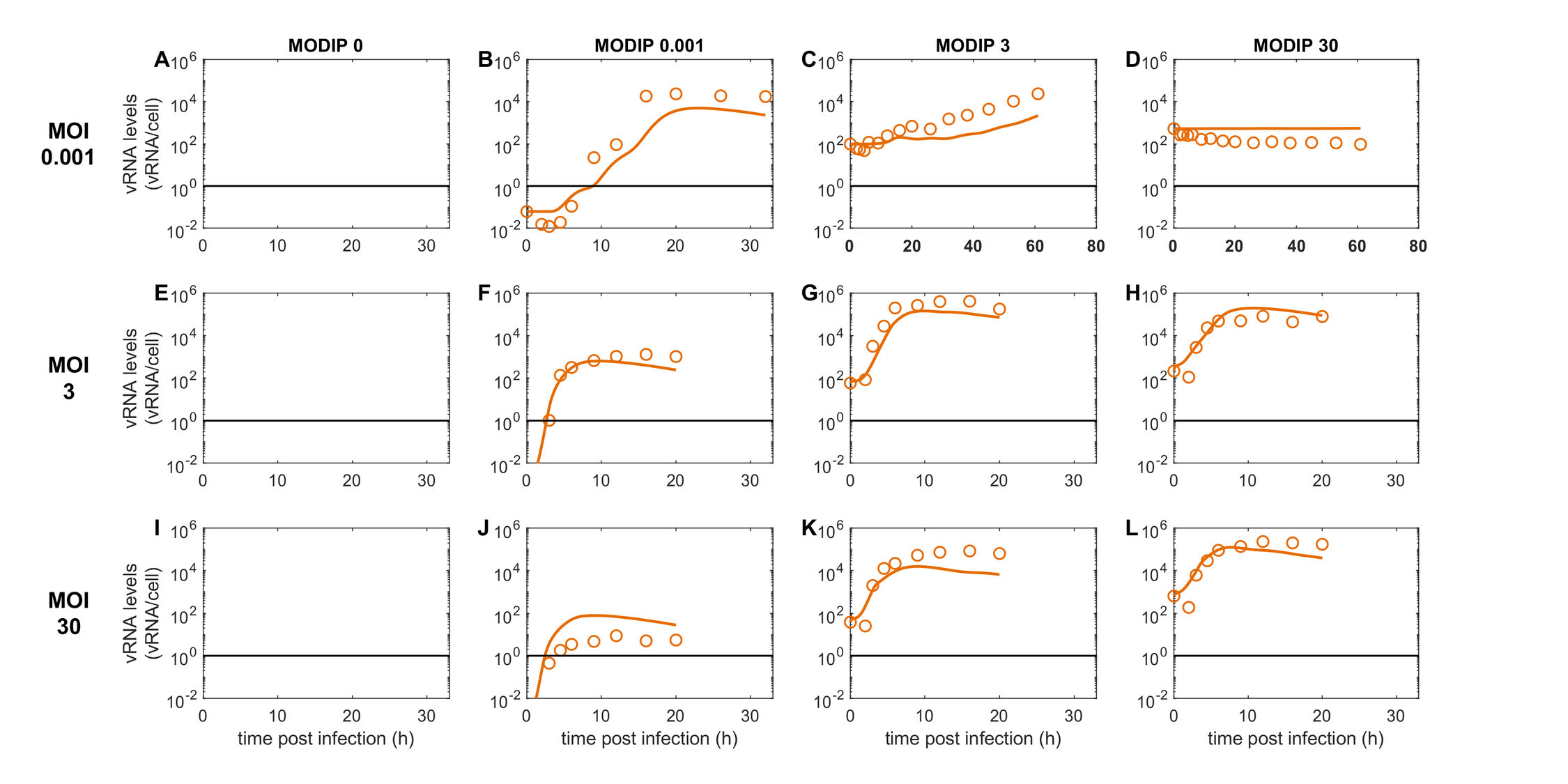

Supplement: S9 Fig — Model fits to measurements of the intracellular levels of DI vRNA for MDCKsus infections with MOI 10−3, 3 and 30 using different MODIPs. (TIF) [file pcbi.1009357.s011.tif]

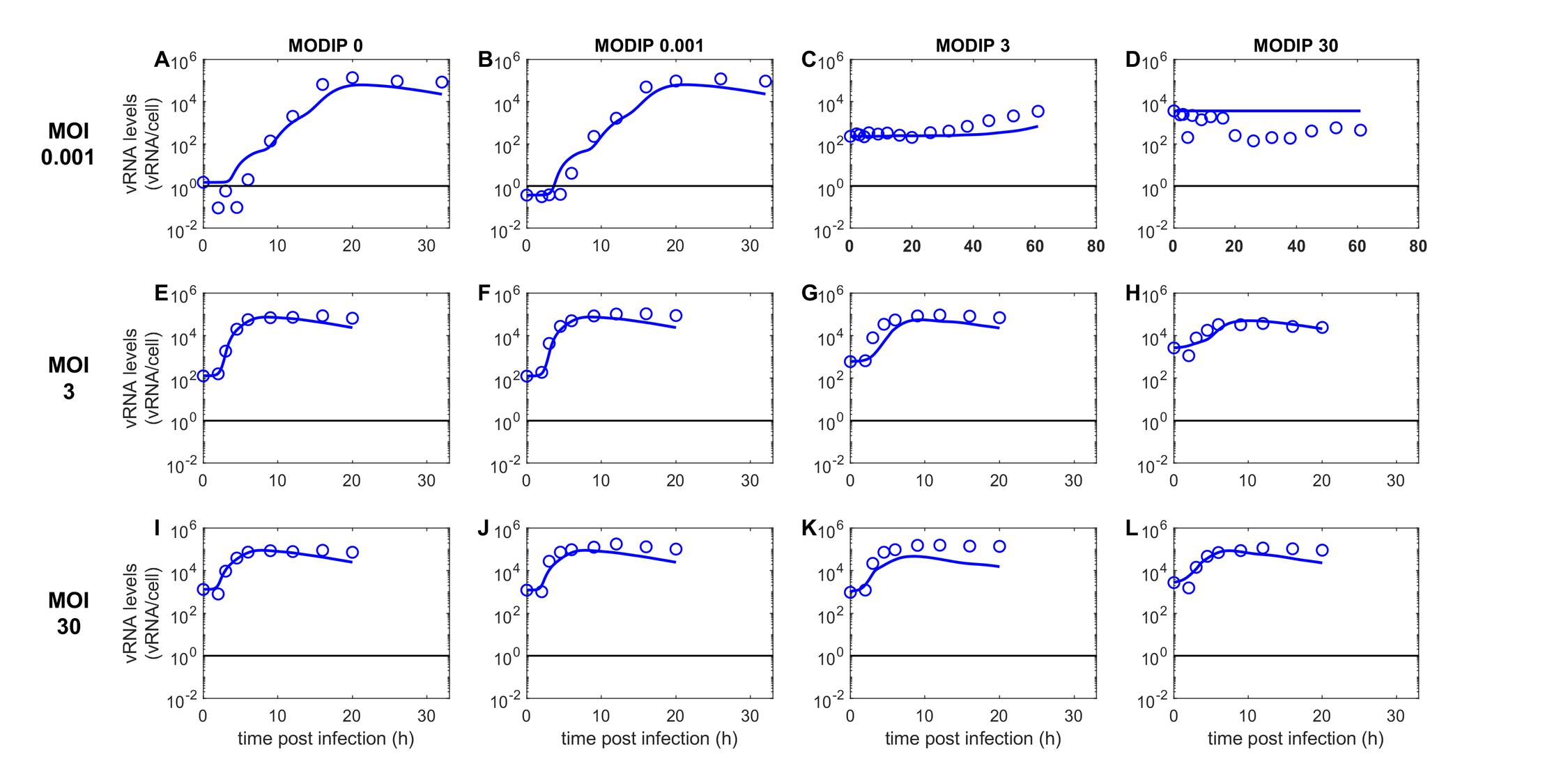

Supplement: S10 Fig — Model fits to measurements of the intracellular levels of segment 5 vRNA for MDCKsus infections with MOI 10−3, 3 and 30 using different MODIPs. (TIF) [file pcbi.1009357.s012.tif]

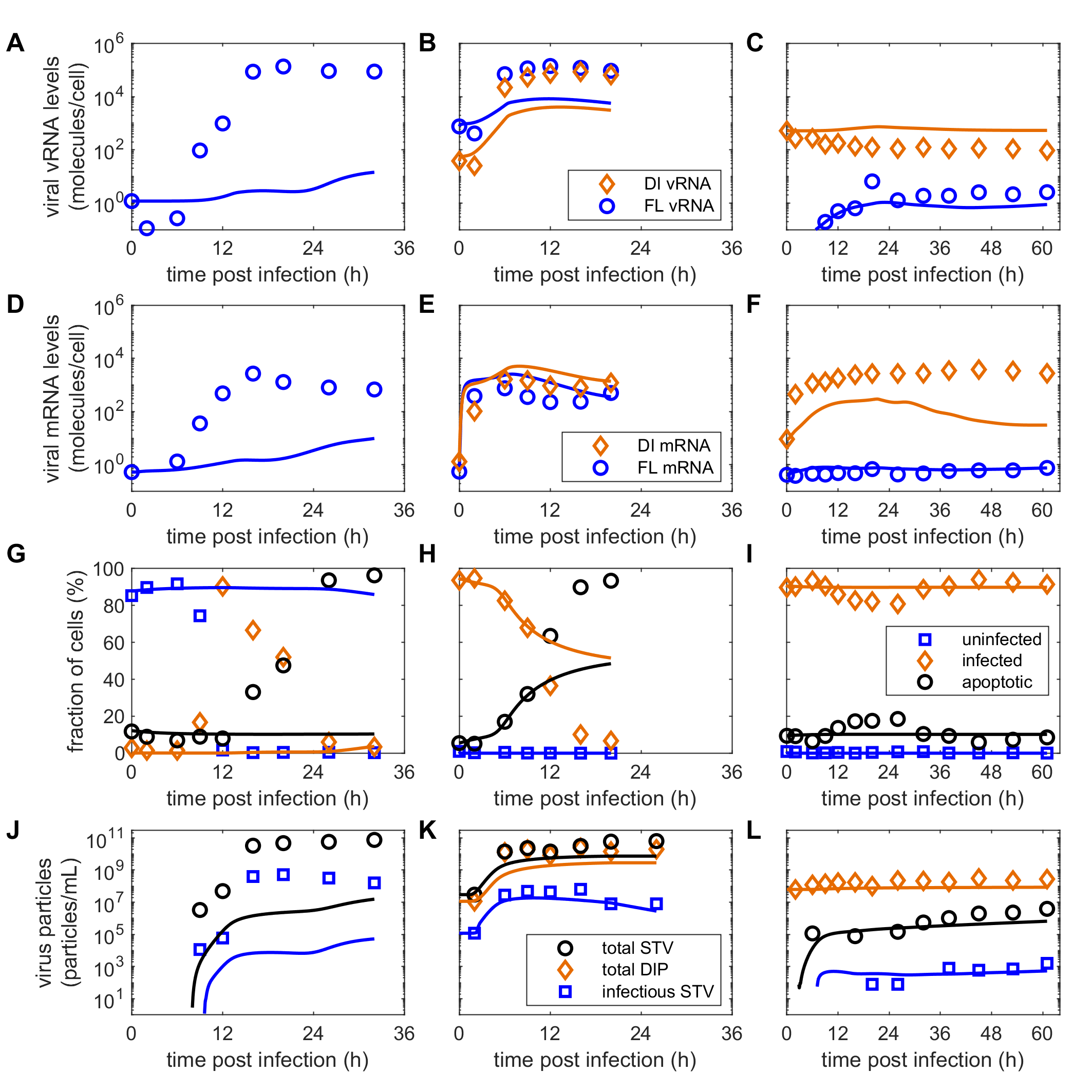

Supplement: S11 Fig — Curves represent model simulations of the basic model calibrated to (A-C) cell-specific vRNA, (D-F) cell-specific viral mRNA, (G-I) cell population and (J-L) virus titer data measured in MDCK suspension cell cultures infected with different amounts of influenza A/PR/8/34 (H1N1) and defective interfering particles (DI244). Results from MOIs and MODIPs of 10−3 and 0 (first column), 30 and 3 (second column), 10−3 and 30 (third column) are shown. The basic model describes IAV and DIP replication and propagation based on Rüdiger et al. [5] and Laske et al. [1] without considering additional model adaptations. (TIF) [file pcbi.1009357.s013.tif]
