# Supplementary material for: Multiscale model of defective interfering particle replication for influenza A virus infection in animal cell culture
Source: PLoS Comput Biol. 2021 Sep 7;17(9):e1009357. doi: 10.1371/journal.pcbi.1009357 (PMC8448327; doi:10.1371/journal.pcbi.1009357)
Supplement: S1 Table — (DOCX) [file pcbi.1009357.s014.docx]

**S1 Table.** **Evaluation of the model fits performed for the basic and the extended model for individual infection conditions.**

| **Condition** | | **SSR** | | **AIC** | |
| --- | --- | --- | --- | --- | --- |
| **MOI** | **MODIP** | **Basic model** | **Extended model** | **Basic model** | **Extended model** |
| 10^-3^ | 0 | 2.0×10^1^ | 6.6×10^0^ | 11.8 | **-90.3** |
| 10^-3^ | 10^-3^ | 2.6×10^1^ | 1.4×10^1^ | -21.3 | **-100.9** |
| 10^-3^ | 3 | 1.6×10^1^ | 2.1×10^1^ | **-282.8** | -231.1 |
| 10^-3^ | 30 | 3.4×10^1^ | 2.9×10^2^ | **-142.9** | 236.8 |
| 3 | 0 | 1.6×10^1^ | 4.5×10^0^ | 18.2 | **-85.4** |
| 3 | 10^-3^ | 6.0×10^1^ | 8.5×10^1^ | **96.0** | 129.7 |
| 3 | 3 | 1.1×10^2^ | 3.2×10^1^ | 155.5 | **15.2** |
| 3 | 30 | 4.1×10^2^ | 1.4×10^2^ | 295.1 | **172.8** |
| 30 | 0 | 1.7×10^1^ | 8.7×10^0^ | 20.6 | **-35.7** |
| 30 | 10^-3^ | 1.9×10^1^ | 1.9×10^2^ | **-4.2** | 214.6 |
| 30 | 3 | 2.4×10^1^ | 1.3×10^1^ | -12.8 | **-80.4** |
| 30 | 30 | 7.3×10^1^ | 3.7×10^1^ | 106.1 | **29.6** |

SSR: sum of squared residuals (errors of each variable were normalized to the respective maximum measurement value);

AIC: Akaike information criterion
