# Supplementary material for: Multiscale model of defective interfering particle replication for influenza A virus infection in animal cell culture
Source: PLoS Comput Biol. 2021 Sep 7;17(9):e1009357. doi: 10.1371/journal.pcbi.1009357 (PMC8448327; doi:10.1371/journal.pcbi.1009357)
Supplement: S2 Table — (DOCX) [file pcbi.1009357.s015.docx]

**S2 Table. Parameters of the intracellular model.**

| **Parameter** | **Description** | **Value** | **Unit** | **Source** |
| --- | --- | --- | --- | --- |
| **** | number of high-affinity binding sites | 150 | sites | [2] |
| **** | number of low-affinity binding sites | 1000 | sites | [2] |
| **** | distance between two adjacent ribosomes | 160 | nucleotides | [8] |
| **$F_{\text{Adv}}$** | DI cRNA replication advantage factor | 0.32 | − | model fit in  S3-S10 Figs |
| **** | fraction of fusion-competent virions | 0.51 | − | [9] |
| **$F_{\text{M}}$** | reduction factor for RdRp-related viral mRNA synthesis | 0.12 | − | model fit in  S3-S10 Figs |
| **$F_{\text{MODIP}}$** | minimum MODIP required to reduce vRNA synthesis | 1.1×10^-3^ | virion·cell^-1^ | vRNA synthesis reduction only observed for MODIPs > 10^-3^ |
| **** | fraction of M2-encoding mRNAs | 0.02 | − | [6] |
| **** | fraction of NEP-encoding mRNAs | 0.125 | − | [10] |
| **** | attachment to high-affinity binding sites | 8.09×10^-2^ | site^-1^·h^-1^ | [6] |
| **** | attachment to low-affinity binding sites | 4.55×10^-4^ | site^-1^·h^-1^ | [6] |
| **** | binding of M1 to nuclear vRNPs | 1×10^-7^ | molecules^-1^·h^-1^ | model fit in  S3-S10 Figs |
| **** | binding of NP to  RdRp-RNA complexes | 3.01×10^-4^ | molecules^-1^·h^-1^ | [9] |
| **** | binding of RdRp-complexes to RNA | 1 | molecules^-1^·h^-1^ | [9] |
| **** | formation rate of vRNP complexes | 1 | molecules^-7^·h^-1^ | [1] |
| **** | degradation of mRNA | 0.33 | h^-1^ | [9] |
| **** | degradation of nascent cRNA/vRNA | 36.36 | h^-1^ | [9] |
| **** | degradation of RNPs | 0.09 | h^-1^ | [9] |
| **** | degradation of RdRp-RNA complexes | 4.25 | h^-1^ | [9] |
| **** | endocytosis | 4.8 | h^-1^ | [9] |
| **** | equilibrium constant of high-affinity sites | 1.13×10^-2^ | sites^-1^ | [2] |
| **Parameter** | **Description** | **Value** | **Unit** | **Source** |
| **** | equilibrium constant of low-affinity sites | 8.33×10^-5^ | sites^-1^ | [2] |
| **** | fusion with endosomes | 58.3 | h^-1^ | model fit in  S3-S10 Figs |
| **** | nuclear import | 6 | h^-1^ | [11] |
| **$K_{\text{R}}$** | mRNA synthesis inhibition constant | 7.8×10^3^ | molecules | model fit in  S3-S10 Figs |
| **** | formation of  RdRp-complexes | 1 | molecules^-2^·h^-1^ | [6] |
| **** | virion release/budding | 6.15×10^3^ | virions·h^-1^ | model fit in  S3-S10 Figs |
| **** | reduction of infectious release | 4.1×10^-4^ | h^-1^ | model fit in  S3-S10 Figs |
| **** | cRNA synthesis | 0.9 | h^-1^ | [5] |
| **** | mRNA synthesis | 1.73×10^5^ | nucleotides·h^-1^ | [5] |
| **** | protein synthesis | 64800 | nucleotides·h^-1^ | [12] |
| **$K_{\text{V}}$** | maximum vRNA synthesis rate | 20.1 | h^-1^ | model fit in  S3-S10 Figs |
| **** | influence of viral components on release | 1.8 | virions | model fit in  S3-S10 Figs |
| **** | length of  segment 1’s mRNA | 2320 | nucleotides | [13] |
| **** | length of  segment 2’s mRNA | 2320 | nucleotides | [13] |
| **** | length of  segment 3’s mRNA | 2211 | nucleotides | [13] |
| **** | length of  segment 4’s mRNA | 1757 | nucleotides | [13] |
| **** | length of  segment 5’s mRNA | 1540 | nucleotides | [13] |
| **** | length of  segment 6’s mRNA | 1392 | nucleotides | [13] |
| **** | length of segment 7’s unspliced mRNA | 1005 | nucleotides | [13] |
| **** | length of segment 8’s unspliced mRNA | 868 | nucleotides | [13] |
| **** | length of DI segment unspliced mRNA | 373 | nucleotides | extrapolated from DI segment vRNA and cRNA length |
| **** | length of segment 1’s vRNA and cRNA | 2341 | nucleotides | [13] |
| **** | length of segment 2’s vRNA and cRNA | 2341 | nucleotides | [13] |
| **** | length of segment 3’s vRNA and cRNA | 2233 | nucleotides | [13] |

| **Parameter** | **Description** | **Value** | **Unit** | **Source** |
| --- | --- | --- | --- | --- |
| **** | length of segment 4’s vRNA and cRNA | 1778 | nucleotides | [13] |
| **** | length of segment 5’s vRNA and cRNA | 1565 | nucleotides | [13] |
| **** | length of segment 6’s vRNA and cRNA | 1413 | nucleotides | [13] |
| **** | length of segment 7’s vRNA and cRNA | 1027 | nucleotides | [13] |
| **** | length of segment 8’s vRNA and cRNA | 890 | nucleotides | [13] |
| **** | length of DI segment vRNA and cRNA | 395 | nucleotides | [17] |
| **** | number of HA molecules in a virion | 500 | molecules·  virion^-1^ | [13] |
| **** | number of NA molecules in a virion | 100 | molecules·  virion^-1^ | [13] |
| **** | number of M1 molecules in a virion | 3000 | molecules·  virion^-1^ | [13] |
| **** | number of M2 molecules in a virion | 40 | molecules·  virion^-1^ | [13] |
| **** | nucleotides bound by one M1 molecule | 200 | nucleotides | [14] |
| **** | nucleotides bound by one NP molecule | 24 | nucleotides | [15] |
| **** | factor connecting the MODIP-to-MOI ratio to the reduction of vRNA synthesis | 5.2 | − | model fit in  S3-S10 Figs |
| **** | exponent connecting the MODIP-to-MOI ratio to the reduction of vRNA synthesis | 0.1 | − | model fit in  S3-S10 Figs |

**Supplementary references**

1. Laske T, Heldt FS, Hoffmann H, Frensing T, Reichl U. Modeling the intracellular replication of influenza A virus in the presence of defective interfering RNAs. Virus Research. 2016;213:90-99.
2. Nunes-Correia I, Ramalho-Santos J, Nir S, de Lima MCP. Interactions of influenza virus with cultured cells: Detailed kinetic modeling of binding and endocytosis. Biochemistry. 1999;38(3): 1095-1101.
3. Rodriguez A, Pérez-González A, Nieto A. Influenza virus infection causes specific degradation of the largest subunit of cellular RNA polymerase II. Journal of Virology. 2007;81(10):5315-5324.
4. Martínez-Alonso M, Hengrung N, Fodor E. RNA-free and ribonucleoprotein-associated influenza virus polymerases directly bind the serine-5-phosphorylated carboxyl-terminal domain of host RNA polymerase II. Journal of Virology. 2016;90(13):6014-6021.
5. Rüdiger D, Kupke SY, Laske T, Zmora P, Reichl U. Multiscale modeling of influenza A virus replication in cell cultures predicts infection dynamics for highly different infection conditions. PLOS Comput Biol. 2019;15(2):e1006819.
6. Heldt FS, Frensing T, Pflugmacher A, Gröpler R, Peschel B, Reichl U. Multiscale modeling of influenza A virus infection supports the development of direct-acting antivirals. PLOS Computational Biology. 2013;9(11): e1003372.
7. Bowling SR, Khasawneh MT, Kaewkuekool S, Cho BR. A logistic approximation to the cumulative normal distribution. Journal of Industrial Engineering and Management. 2009;2(1):114-127.
8. Arava Y, Wang YL, Storey JD, Liu CL, Brown PO, Herschlag D. Genome-wide analysis of mRNA translation profiles in Saccharomyces cerevisiae. Proceedings of the National Academy of Sciences of the United States of America. 2003;100: 3889-3894.
9. Heldt FS, Frensing T, Reichl U. Modeling the intracellular dynamics of influenza virus replication to understand the control of viral RNA synthesis. Journal of Virology. 2012;86(15): 7806-7817.
10. Robb NC, Jackson D, Vreede FT, Fodor E. Splicing of influenza A virus NS1 mRNA is independent of the viral NS1 protein. Journal of General Virology. 2010;91: 2331-2340.
11. Babcock HP, Chen C, Zhuang XW. Using single-particle tracking to study nuclear trafficking of viral genes. Biophysical Journal. 2004;87: 2749-2758.
12. Spirin, AS. Ribosome structure and protein biosynthesis. The Benjamin/Cummings Publishing Company. 1986.
13. Lamb RA, Krug RM. Orthomyxoviridae: the viruses and their replication. In: Knipe DM, Howley PM, Griffin EG, editors. Fields virology, 4th edition. Lippincott Williams & Wilkins; 2001. p.1487-153.1
14. Wakefield L, Brownlee GG. Rna-Binding Properties of Influenza-a Virus Matrix Protein M1. Nucleic Acids Research. 1989;17: 8569-8580.
15. Portela A, Digard P. The influenza virus nucleoprotein: a multifunctional RNA-binding protein pivotal to virus replication. Journal of General Virology. 2002;83: 723-734.
16. Schulze-Horsel J, Schulze M, Agalaridis G, Genzel Y, Reichl U. Infection dynamics and virus-induced apoptosis in cell culture-based influenza vaccine production-Flow cytometry and mathematical modeling. Vaccine. 2009;27: 2712-2722.
17. Dimmock NJ, Rainsford EW, Scott PD, Marriott AC. Influenza virus protecting RNA: an effective prophylactic and therapeutic antiviral. Journal of Virology. 2008;82(17):8570-8578.
