# Supplementary material for: Multiscale model of defective interfering particle replication for influenza A virus infection in animal cell culture
Source: PLoS Comput Biol. 2021 Sep 7;17(9):e1009357. doi: 10.1371/journal.pcbi.1009357 (PMC8448327; doi:10.1371/journal.pcbi.1009357)
Supplement: S3 Table — (DOCX) [file pcbi.1009357.s016.docx]

**S3 Table. Parameters of the cell population model.**

| **Parameter** | **Description** | **Value** | **Unit** | **Source** |
| --- | --- | --- | --- | --- |
| **** | maximum cell growth rate | 0.03 | h^-1^ | [16] |
| **** | number of high-affinity binding sites | 150 | sites·cell^-1^ | [2] |
| **** | number of low-affinity binding sites | 1000 | sites·cell^-1^ | [2] |
| **** | ratio of infected cells to fused virions | 1 | cells·virion^-1^ | [6] |
| **** | cell growth reduction in very high virus concentrations | 0.63 | - | model fit in  S3-S10 Figs |
| **$K_{\text{I}}$** | maximum apoptosis rate of infected cell | 0.27 | h^-1^ | model fit in  S3-S10 Figs |
| **** | apoptosis rate of uninfected cells | 1.2×10^-2^ | h^-1^ | model fit in  S3-S10 Figs |
| **** | attachment to high-affinity binding sites | 3.32×10^-8^ | mL·sites^-1^·h^-1^ | [6] |
| **** | attachment to low-affinity binding sites | 1.85×10^-10^ | mL·sites^-1^·h^-1^ | [6] |
| **** | degradation/clearance of infectious virions | 0.2 | h^-1^ | adjusted to infectious titer reduction observed in experiments |
| **** | endocytosis | 4.8 | h^-1^ | [9] |
| **** | equilibrium constant of high-affinity sites | 4.48×10^-9^ | mL·sites^-1^ | [2] |
| **** | equilibrium constant of low-affinity sites | 3.32×10^-11^ | mL·sites^-1^ | [2] |
| **** | fusion with endosomes | 58.3 | h^-1^ | model fit in  S3-S10 Figs |
| **** | lysis of apoptotic cells | 0.16 | h^-1^ | model fit in  S3-S10 Figs |
| **** | maximum cell concentration | 1×10^7^ | cells·mL^-1^ | maximum cell concentration observed in control flasks |
| **** | time after cell infection at which the rate of virus-induced apoptosis reaches its half-maximum | 6.65 | h | model fit in  S3-S10 Figs |
| **** | distribution factor of the virus-induced apoptosis rate | 1.7 | h^-1^ | fixed to value inducing a normal distribution |

**Supplementary references**

1. Laske T, Heldt FS, Hoffmann H, Frensing T, Reichl U. Modeling the intracellular replication of influenza A virus in the presence of defective interfering RNAs. Virus Research. 2016;213:90-99.
2. Nunes-Correia I, Ramalho-Santos J, Nir S, de Lima MCP. Interactions of influenza virus with cultured cells: Detailed kinetic modeling of binding and endocytosis. Biochemistry. 1999;38(3): 1095-1101.
3. Rodriguez A, Pérez-González A, Nieto A. Influenza virus infection causes specific degradation of the largest subunit of cellular RNA polymerase II. Journal of Virology. 2007;81(10):5315-5324.
4. Martínez-Alonso M, Hengrung N, Fodor E. RNA-free and ribonucleoprotein-associated influenza virus polymerases directly bind the serine-5-phosphorylated carboxyl-terminal domain of host RNA polymerase II. Journal of Virology. 2016;90(13):6014-6021.
5. Rüdiger D, Kupke SY, Laske T, Zmora P, Reichl U. Multiscale modeling of influenza A virus replication in cell cultures predicts infection dynamics for highly different infection conditions. PLOS Comput Biol. 2019;15(2):e1006819.
6. Heldt FS, Frensing T, Pflugmacher A, Gröpler R, Peschel B, Reichl U. Multiscale modeling of influenza A virus infection supports the development of direct-acting antivirals. PLOS Computational Biology. 2013;9(11): e1003372.
7. Bowling SR, Khasawneh MT, Kaewkuekool S, Cho BR. A logistic approximation to the cumulative normal distribution. Journal of Industrial Engineering and Management. 2009;2(1):114-127.
8. Arava Y, Wang YL, Storey JD, Liu CL, Brown PO, Herschlag D. Genome-wide analysis of mRNA translation profiles in Saccharomyces cerevisiae. Proceedings of the National Academy of Sciences of the United States of America. 2003;100: 3889-3894.
9. Heldt FS, Frensing T, Reichl U. Modeling the intracellular dynamics of influenza virus replication to understand the control of viral RNA synthesis. Journal of Virology. 2012;86(15): 7806-7817.
10. Robb NC, Jackson D, Vreede FT, Fodor E. Splicing of influenza A virus NS1 mRNA is independent of the viral NS1 protein. Journal of General Virology. 2010;91: 2331-2340.
11. Babcock HP, Chen C, Zhuang XW. Using single-particle tracking to study nuclear trafficking of viral genes. Biophysical Journal. 2004;87: 2749-2758.
12. Spirin, AS. Ribosome structure and protein biosynthesis. The Benjamin/Cummings Publishing Company. 1986.
13. Lamb RA, Krug RM. Orthomyxoviridae: the viruses and their replication. In: Knipe DM, Howley PM, Griffin EG, editors. Fields virology, 4th edition. Lippincott Williams & Wilkins; 2001. p.1487-153.1
14. Wakefield L, Brownlee GG. Rna-Binding Properties of Influenza-a Virus Matrix Protein M1. Nucleic Acids Research. 1989;17: 8569-8580.
15. Portela A, Digard P. The influenza virus nucleoprotein: a multifunctional RNA-binding protein pivotal to virus replication. Journal of General Virology. 2002;83: 723-734.
16. Schulze-Horsel J, Schulze M, Agalaridis G, Genzel Y, Reichl U. Infection dynamics and virus-induced apoptosis in cell culture-based influenza vaccine production-Flow cytometry and mathematical modeling. Vaccine. 2009;27: 2712-2722.
17. Dimmock NJ, Rainsford EW, Scott PD, Marriott AC. Influenza virus protecting RNA: an effective prophylactic and therapeutic antiviral. Journal of Virology. 2008;82(17):8570-8578.
