# Supplementary material for: Multiscale model of defective interfering particle replication for influenza A virus infection in animal cell culture
Source: PLoS Comput Biol. 2021 Sep 7;17(9):e1009357. doi: 10.1371/journal.pcbi.1009357 (PMC8448327; doi:10.1371/journal.pcbi.1009357)
Supplement: S4 Table — (DOCX) [file pcbi.1009357.s017.docx]

**S4 Table. Sensitivity of intracellular and cell population model parameters.**

| **Parameter** | **Local sensitivity (%),**  **intracellular model output** | **Local sensitivity (%),**  **cell population model output** |
| --- | --- | --- |
| **** | 3.0×10^-6^ | 2.4×10^-6^ |
| **** | 1.0×10^-7^ | 1.2×10^-7^ |
| **** | 1.5×10^-4^ | 1.0×10^-5^ |
| **$F_{\text{Adv}}$** | 2.5×10^-4^ | 1.3×10^-4^ |
| **** | 3.5×10^-5^ | 1.9×10^-5^ |
| **$F_{\text{M}}$** | 2.3×10^-4^ | 1.0×10^-5^ |
| **** | 2.1×10^-7^ | 6.4×10^-9^ |
| **** | 2.5×10^-6^ | 9.2×10^-7^ |
| **** | 1.4×10^-7^ | 6.3×10^-8^ |
| **** | 2.2×10^-8^ | 2.5×10^-8^ |
| **** | 7.4×10^-5^ | 1.5×10^-5^ |
| **** | 6.0×10^-6^ | 2.8×10^-6^ |
| **** | 1.4×10^-7^ | 6.8×10^-8^ |
| **** | 2.3×10^-11^ | 2.7×10^-12^ |
| **** | 2.0×10^-5^ | 1.1×10^-6^ |
| **** | 1.7×10^-7^ | 8.3×10^-8^ |
| **** | 8.4×10^-5^ | 1.9×10^-5^ |
| **** | 1.4×10^-5^ | 2.3×10^-6^ |
| **** | 4.9×10^-6^ | 3.9×10^-6^ |
| **** | 3.3×10^-7^ | 1.6×10^-7^ |
| **** | 1.9×10^-8^ | 2.2×10^-8^ |
| **** | 1.7×10^-7^ | 3.2×10^-7^ |
| **** | 5.8×10^-7^ | 2.8×10^-7^ |
| **$K_{\text{R}}$** | 5.0×10^-5^ | 5.5×10^-6^ |
| **** | 3.9×10^-11^ | 2.2×10^-11^ |
| **** | 1.4×10^-4^ | 2.4×10^-4^ |
| **** | 1.8×10^-9^ | 1.2×10^-9^ |
| **** | 9.9×10^-4^ | 3.5×10^-4^ |
| **** | 9.3×10^-5^ | 1.0×10^-5^ |
| **** | 1.5×10^-4^ | 1.0×10^-5^ |
| **$K_{\text{V}}$** | 2.9×10^-3^ | 1.0×10^-3^ |
| **** | 2.9×10^-9^ | 1.0×10^-9^ |
| **** | 2.7×10^-4^ | 9.9×10^-6^ |
| **** | 9.5×10^-5^ | 2.2×10^-6^ |
| **** | 7.9×10^-11^ | 3.4×10^-11^ |
| **** | 1.6×10^-11^ | 4.3×10^-12^ |
| **Parameter** | **Local sensitivity (%),**  **intracellular model output** | **Local sensitivity (%),**  **cell population model output** |
| **** | 9.7×10^-4^ | 1.9×10^-5^ |
| **** | 2.0×10^-11^ | 4.9×10^-12^ |
| **** | 4.9×10^-4^ | 1.5×10^-5^ |
| **** | 2.5×10^-6^ | 9.1×10^-7^ |
| **** | 2.9×10^-5^ | 1.1×10^-11^ |
| **** | 1.6×10^-5^ | 2.6×10^-7^ |
| **** | 2.3×10^-5^ | 3.2×10^-7^ |
| **** | 2.1×10^-5^ | 2.9×10^-7^ |
| **** | 1.4×10^-5^ | 1.9×10^-7^ |
| **** | 1.1×10^-5^ | 1.4×10^-7^ |
| **** | 8.6×10^-6^ | 1.2×10^-7^ |
| **** | 4.5×10^-6^ | 6.2×10^-8^ |
| **** | 3.4×10^-6^ | 4.7×10^-8^ |
| **** | 1.1×10^-6^ | 4.1×10^-9^ |
| **** | 1.5×10^-11^ | 3.7×10^-12^ |
| **** | 1.5×10^-11^ | 4.0×10^-12^ |
| **** | 2.3×10^-4^ | 3.8×10^-6^ |
| **** | 2.7×10^-11^ | 1.2×10^-11^ |
| **** | 5.4×10^-7^ | 3.5×10^-10^ |
| **** | 8.2×10^-4^ | 1.0×10^-5^ |
| **** | 2.1×10^-3^ | 8.1×10^-4^ |
| **** | 1.6×10^-3^ | 6.8×10^-4^ |
| **** | 8.2×10^-6^ | 1.5×10^-5^ |
| **** | 3.3×10^-4^ | 1.7×10^-4^ |
| **** | 1.6×10^-6^ | 4.4×10^-6^ |
| **$K_{\text{I}}$** | 1.0×10^-3^ | 3.4×10^-4^ |
| **** | 3.0×10^-6^ | 2.0×10^-5^ |
| **** | 2.6×10^-6^ | 2.3×10^-6^ |
| **** | 3.8×10^-9^ | 3.2×10^-9^ |
| **** | 4.1×10^-6^ | 2.9×10^-5^ |
| **** | 7.7×10^-7^ | 9.1×10^-7^ |
| **** | 9.3×10^-10^ | 7.8×10^-10^ |
| **** | 4.1×10^-5^ | 4.5×10^-5^ |
| **** | 1.2×10^-6^ | 1.3×10^-6^ |
| **** | 1.6×10^-3^ | 8.5×10^-4^ |
| **** | 5.0×10^-7^ | 1.0×10^-6^ |
