# Supplementary material for: Multiscale model of defective interfering particle replication for influenza A virus infection in animal cell culture
Source: PLoS Comput Biol. 2021 Sep 7;17(9):e1009357. doi: 10.1371/journal.pcbi.1009357 (PMC8448327; doi:10.1371/journal.pcbi.1009357)
Supplement: S5 Table — (DOCX) [file pcbi.1009357.s018.docx]

**S5 Table: Primers used for real-time RT qPCR of mRNA.**

| **Target** | **Primer name** | **Sequence (5´→3´)** |
| --- | --- | --- |
| Segment 1 | Seg 1 Realtime for | GAAGACCCAGATGAAGGC |
| DI244 | DI244 Realtime for | GAAGACAGGAGAAGACTGAG |
