# Supplementary material for: Multiscale model of defective interfering particle replication for influenza A virus infection in animal cell culture
Source: PLoS Comput Biol. 2021 Sep 7;17(9):e1009357. doi: 10.1371/journal.pcbi.1009357 (PMC8448327; doi:10.1371/journal.pcbi.1009357)
Supplement: S6 Table — (DOCX) [file pcbi.1009357.s019.docx]

**S6 Table: Primers used for reference standard generation of mRNA.**

| **Target** | **Primer name** | **Sequence (5´→3´)** |
| --- | --- | --- |
| Segment 1 and DI244 | Seg 1 Uni T7 for | TAATACGACTCACTATAGGGAGCGAAAGCAGGTCAATTAT |
|  | PR8 Seg 1 dT rev | TTTTTTTTTTTTTTTTAAACTATTCGA |
