# Supplementary material for: Multiscale model of defective interfering particle replication for influenza A virus infection in animal cell culture
Source: PLoS Comput Biol. 2021 Sep 7;17(9):e1009357. doi: 10.1371/journal.pcbi.1009357 (PMC8448327; doi:10.1371/journal.pcbi.1009357)
Supplement: Attachment — Submitted filename: Review-5-21.pdf [file pcbi.1009357.s020.pdf]

This article presents findings from both in vitro experiments and modeling to provide further understanding on the interaction between DIPs and STVs with the focus on the potential therapeutic use for DIPs. The paper was thorough with well-reasoned logic behind both the experiments and the modeling choices. The authors clearly referenced relative studies and parameters and their modeling results were in accordance with previous work. This manuscript is clearly well thought out and presents new and useful information along with suggestions for further research.

A suggestion for improvement is that as models are changed or added to they are described as having better fits and the parameters estimated are given in either Table 1 or the Supplemental Information. I would like to see, if possible, a quantitative comparison of model fits, potentially with an AIC score, in order to make this statement more robust.

Besides that point, some minor observations are:

1. For Figure 1 caption: Explain the symbols for STV and DIP given in Fig1A as they show up later in Fig3.
2. Lines 209-211: "*… expands their scope by including observed viral RNA replication phenomena and DIP propagation in cell culture.*" It is not obvious if the observations are from the experiments done previously in this paper or not. I think it would clarity if that information was given here rather than a few sentences later.
3. Fig 3: If possible, I would like to see some of the parameters, at least on A, to help familiarize the reader with the model before seeing the equations. It may help orient the reader even if only considering a simplified version of the model.
4. Line 252" "*..to re-evaluate the initial conditions…"* I suggest clarifying if the IC being re-evaluated are intra- or extra-cellular or both.
5. Line 257: "*…cells showing overall..*" May read better as "…*cells which show overall…*"
6. Lines 268-272: I suggest adding a sentence explaining why a set of parameters could not be found. Maybe as simple as mentions that biological systems and their interactions are complex.
7. Fig 4: To aid in clarity, adding column titles for to separate out the figures may be helpful. Perhaps as simple as adding "Basic Model" over A and "Extended Model" over B. Then a line separating out E and F as it does not apply to them. Not necessary but potential useful.
8. SI Appendix 1: Potentially merely a formatting issue on my particular computer but supplemental equations were not numbered.
