# Supplementary material for: Multiscale model of defective interfering particle replication for influenza A virus infection in animal cell culture
Source: PLoS Comput Biol. 2021 Sep 7;17(9):e1009357. doi: 10.1371/journal.pcbi.1009357 (PMC8448327; doi:10.1371/journal.pcbi.1009357)
Supplement: Attachment — Submitted filename: Ruediger_ReviewerResponse.docx [file pcbi.1009357.s021.docx]

**MAX-PLANCK-INSTITUTE**

**FOR DYNAMICS OF COMPLEX TECHNICAL SYSTEMS MAGDEBURG**

BIOPROCESS ENGINEERING

Sandtorstrasse 1 I 39106 Magdeburg I Germany

**Daniel Rüdiger, M. Sc.**

Doctoral Researcher

BIOPROCESS ENGINEERING

Sandtorstrasse 1

39106 Magdeburg

Germany

**P**+49 391 6110-206

ruedigerd@mpi-magdeburg.mpg.de

www.mpi-magdeburg.mpg.de

Magdeburg, 25.06.2021

**Response to Reviewer Comments**

Dear Editors,

We hereby submit the revision of our manuscript “Multiscale model of defective interfering particle replication for influenza A virus infection in animal cell culture“ for publication in PLOS Computational Biology. Please find our detailed response to the reviewer comments below.

**Response to Reviewer #1**

*This article presents findings from both in vitro experiments and modeling to provide further understanding on the interaction between DIPs and STVs with the focus on the potential therapeutic use for DIPs. The paper was thorough with well-reasoned logic behind both the experiments and the modeling choices. The authors clearly referenced relative studies and parameters and their modeling results were in accordance with previous work. This manuscript is clearly well thought out and presents new and useful information along with suggestions for further research.*

*A suggestion for improvement is that as models are changed or added to they are described as having better fits and the parameters estimated are given in either Table 1 or the Supplemental Information. I would like to see, if possible, a quantitative comparison of model fits, potentially with an AIC score, in order to make this statement more robust.*

Based on the reviewer’s suggestion, we have calculated the AIC values for individual experiments and the complete data set. The results are presented in S1 Table and referenced in line 355-363 (all references to lines refer to the marked-up copy).

*Besides that point, some minor observations are:*

*1. For Figure 1 caption: Explain the symbols for STV and DIP given in Fig1A as they show up later in Fig3.*

We have added an explanation of the symbols for STVs and DIPs to Figure 1.

*2. Lines 209-211: “... expands their scope by including observed viral RNA replication phenomena and DIP propagation in cell culture.” It is not obvious if the observations are from the experiments done previously in this paper or not. I think it would clarity if that information was given here rather than a few sentences later.*

In agreement with the reviewer’s suggestion, we clarified that we are referring to experiments performed in our study in line 212-213.

*3. Fig 3: If possible, I would like to see some of the parameters, at least on A, to help familiarize the reader with the model before seeing the equations. It may help orient the reader even if only considering a simplified version of the model.*

We have included model parameters used on the cell population level in Figure 3.

*4. Line 252” “..to re-evaluate the initial conditions...” I suggest clarifying if the IC being re-evaluated are intra- or extra-cellular or both.*

Following the reviewer’s suggestion, we expanded the description of the initial conditions used for the simulation of the intracellular level in line 256-260.

*5. Line 257: “...cells showing overall..” May read better as “...cells which show overall...”*

We agree with the reviewer and have changed the wording in line 265.

*6. Lines 268-272: I suggest adding a sentence explaining why a set of parameters could not be found. Maybe as simple as mentions that biological systems and their interactions are complex.*

In agreement with the reviewer’s suggestion, we included an additional sentence that links the problem to identify suitable parameters for the basic model to the complexity of STV and DIP co-infection in line 275-277.

*7. Fig 4: To aid in clarity, adding column titles for to separate out the figures may be helpful. Perhaps as simple as adding “Basic Model” over A and “Extended Model” over B. Then a line separating out E and F as it does not apply to them. Not necessary but potential useful.*

We agree with the reviewer and have modified Figure 4 according to this suggestion.

*8. SI Appendix 1: Potentially merely a formatting issue on my particular computer but supplemental equations were not numbered.*

The numbering of equations in S1 Appendix was implemented via MathType and is displayed as intended using Microsoft Word on our PC. We will check this issue again in the galley proof.

**Response to Reviewer #2**

*The work by Rudiger et al. investigates the dynamic impacts of a defective interfering particle (DIP) of influenza A virus (IAV) on the standard IAV virus (STV) using a combination of experimental and mathematical modeling approach. The authors infected MDCK cells suspension cells using 12 different combinations of MOIs for STV and DIPs (MODIP is used to denote MOIs for DIPs), and measured a variety of quantities of interests including levels of cell deaths, vRNA, viral mRNA, total virus yield and infectious virus yield. This data set suggests that DIP can effectively inhibit STV replication and prevent cell death when the ratio of MODIP over MOI is very high (e.g. >1000). The authors further developed a multiscale mathematical model considering viral replication in a cell, and viral spread between cells. This model is an extension of models developed previously by the authors. Fitting the model to the experimental data, the authors identified key intracellular mechanisms that regulate transcription of different gene segments of IAV and the DIP. Finally, model simulations revealed conditions under which DIP would be able to completely inhibit STV growth in cell culture and conditions allowing for maximum DIP production.*

*Overall, I found this work very interesting, well designed and executed. First of all, DIP has been proposed to be a promising therapeutic approach against novel viral outbreaks. Understanding the dynamic impacts of DIPs on STV is key to the development of this approach into clinical use. Therefore, this work addresses an important question. Second, the data presented here are novel in that it explores how DIP impacts on STV under different MOIs. A high MOI is required for the survival of DIP and thus the ability of DIP to inhibit STV. The works provides a perfect dataset to quantify how effectiveness of DIP is quantitatively related to MOI. Third, the application of the multiscale model framework to understand the datasets is well designed and executed. Although the model is very complicated and it is not clear to me whether such a complicated model is absolutely necessary, using such a model is extremely useful given the highly nonlinear interactions between DIP and STV and the highly complex patterns in the dataset. Lastly, the work is well written. Therefore, I applaud the authors effort to perform the interesting experiments and using the multiscale framework to interpret the patterns of the datasets.*

*I do not have any major concerns; here are some minor concerns that need to be addressed.*

*First, the conclusions from the model development and model fitting section seem to be made based on visual inspections, rather than rigorous statistical tests (at least from what is written). I think it will be important to do or present quantitative analyses of the model (especially for PLOS CB). For example, it would be useful to know how many ODEs and parameter values there are in the multiscale model. The goodness of fits or AIC values of the basic model and extended model.*

According to the reviewer’s suggestion, we have calculated the SSR and AIC values, which are presented in S1 Table. In line 355-363 (all references to lines refer to the marked-up copy), we address these results and also report the number of ODEs and parameters for the basic and the extended model.

*Second, I think it would be interesting and important to test how sensitive the fit of the model to data with respect to changes in the fixed parameter values in the model. This will allow one to see which part of the (complicated) model is important in explaining the data.*

Following the reviewer’s suggestion, we conducted a parameter sensitivity analysis and included the results in S4 Table. The calculations were performed according to Heldt et al. (<https://doi.org/10.1128/jvi.00080-12>) and are mentioned in line 756-758.

*Third, it would be interesting to put the results of the works, e.g. the role of MOI on effectiveness of DIP, into the context of existing literature (in the Discussion perhaps). For example, the recent work by Martin et al. (*[*https://doi.org/10.1371/journal.ppat.1008974*](https://doi.org/10.1371/journal.ppat.1008974)*) showed MOI of the STV has large impacts on the dynamics of STV replication and the interferon responses. It would be interesting to know how the data/results of the work here is related to Martin et al.*

Based on the reviewer’s feedback, we added a paragraph discussing the effect of the MOI on STV and DIP production in line 463-474. There, we also address the results of Martin et al.

**Have the authors made all data and (if applicable) computational code underlying the findings in their manuscript fully available?**

*Reviewer #1: Yes*

*Reviewer #2: None*

The experimental data are provided in S1 Data. The source code for model calibration and simulation is available at <https://github.com/ModIAV/STV_DIP_Coinfection>.

**Additional changes to the manuscript**

Update of the confidence intervals by including more bootstrap iterations:

Table 1, Line 382

Expansion of the discussion including a recently published article (Phan et al., <https://doi.org/10.1128/jvi.02102-20>):

Lines 492-505

Adjustment of the wording to improve clarity:

Lines 42-43, 157, 211, 253, 263-265, 267, 296, 340-341, 459, 515, 528, 530, 547-553, 556-558, 562-565, 588, 593-594, 726, 758-760, 830-832, 1071-1072

Adjustment of spaces and line breaks:

Lines 82, 104, 164, 291, 335, 436-437

Adjustment of inconsistent formatting:

Lines 148, 349, 511, 756, 858, 860, 889, 967, 1056

**Changes to the reference list**

Reference #15 (Hein et al.), which was cited as a forthcoming article in the original submission, has been published and was updated accordingly.

Reference #33 (Phan et al.) was added as it provides experimental data about co-infection dynamics using two DIPs containing deletions in different segments (segment 2 and 4). This paper was published during the review process of our manuscript and provides important insights into a topic that we discussed in the original submission.

Reference #37 (Akaike) was added to accompany the calculation of the Akaike information criterion suggested by the reviewers.

References #40-42 (Martin et al., Aggarwal et al., Isken et al.) were added to discuss the effect of the MOI on STV and DIP production suggested by reviewer #2.

Reference #60 (Heldt et al.) was added to accompany the calculation of the parameter sensitivity suggested by reviewer #2.

Yours sincerely,

Daniel Rüdiger
